# Endocrine-Disrupting Chemicals: A Looming Threat to Current and Future Generations

**DOI:** 10.3390/ijms25158222

**Published:** 2024-07-27

**Authors:** Sergio A. Cortes-Ramirez, Shuk-Mei Ho, Yuet-Kin Leung

**Affiliations:** 1 Department of Pharmacology and Toxicology, Winthrop P. Rockefeller Cancer Institute, University of Arkansas for Medical Sciences, Little Rock, AR 72205, USA; cortessergioa@uams.edu; 2 Central Arkansas Veterans Healthcare System, Little Rock, AR 72205, USA

With rapid modernization, environmental pollutants have become a major concern for human health, contributing to diseases such as asthma, cardiovascular diseases, obesity, infertility, and cancers. One major class of pollutants affecting current and future human health is endocrine-disrupting chemicals (EDCs).

EDCs are exogenous compounds, either natural or synthetic, that disrupt the synthesis, secretion, transport, metabolism, binding, action, and elimination of natural hormones. Initially, EDCs were thought to act only through ligand agonism and antagonism, but now, over ten molecular mechanisms have been identified. These include the disruption of ligand–receptor interaction, dysregulation of receptor expression, interference of signal transduction, alteration of epigenetic landscapes, interruption of hormone synthesis, transport, distribution, and clearance, and the modification of cell fate. These diverse actions of the EDCs may explain their broad impact on health and disease [1]. Moreover, there is wide complexity in each one of these mechanisms. A clear example is epigenetic disruption, where previous evidence described the effects of EDCs on main epigenetic mediators such as histone mark modifications, DNA methylation, and non-coding RNA expression, which might impact early development and imprinting, as well as across generations, explaining the alarming increase in the occurrence of some diseases [2,3,4].

Due to their diverse mechanisms of action, characterizing EDCs remains a major challenge in modern toxicology. Unlike traditional toxicants that follow a linear dose–response pattern (a high dose with strong responses), EDCs have various effects depending on the dose, the timing of exposure, and their signaling targets. Therefore, EDCs exhibit non-monotonic, biphasic, or multiphasic dose responses, as shown in multiple in vitro and in vivo models. For these reasons, traditional toxicological principles may not apply to studying EDCs. Current toxicological approaches need updating to include specific tests for assessing low-dose effects of EDCs. This may involve incorporating molecular tests known for their sensitivities for low doses in cell, tissue-based, and whole animal studies.

In this Special Issue “Endocrine Disruption and Human Diseases 2.0”, we feature nine articles: five original research papers and four review articles. These articles illuminate how EDCs disrupt signaling pathways, epigenetic programming, and molecular mechanisms linked to human diseases such as obesity, infertility, behavioral disorders, and cancer. Several articles explore the developmental origins of health and disease (DOHaD) effect, as well as the potential for transgenerational inheritance. Overall, this issue’s breadth and depth shed new light on the causal links between low-dose EDC exposure and human diseases.

Some environmental obesogenic chemicals are well-characterized EDCs and they are linked to a decline in male fertility across generations. These chemicals disrupt energy metabolism in Sertoli cells, which are crucial for sperm development. One EDC, tributyltin (TBT), interferes with a key transcription factor called peroxisome proliferator-activated receptor γ (PPARγ) in these cells, hindering their ability to nourish sperm. This disrupts the delicate metabolic cooperation between testicular cells and sperm function. The effects can even be passed down to future generations. Sousa et al. reviewed literature in this area and proposed using a new approach known as adverse outcome pathways (AOPs) to examine these effects and develop regulations to protect male fertility from obesogenic EDCs [5].

Another EDC that affects testicular function is bisphenol A (BPA). Varma et al. exposed pregnant rats to BPA and found that it affects testicular long-chain fatty acid metabolism in the adult testes of the offspring. Interestingly, a BPA replacement, bisphenol S BPS, did not elicit similar effects. Arachidonic acid (20:4 n-6, ARA) and docosapentaenoic acid (22:5 n-6, DPA) levels were decreased, along with expression of peroxisome proliferator-activated receptor α (PPARα), PPARγ proteins, and Cation Channel Sperm Associated 2 CATSPER2 mRNA in the BPA-exposed testis. These molecular and cellular regulators, in turn, control sperm maturation and motility. These findings suggest that early-life (gestation) exposure to specific bisphenols can affect sperm development in adulthood [6].

Kolan et al. reviewed the effect of EDC exposure on reproductive health, specifically focusing on preterm birth (PTB), the leading cause of death in children under five. Their systematic review of literature published in the last 20 years examined the association between common EDCs (BPA, phthalates, organochlorine/organophosphate pesticides, lead, and Polybrominated Diphenyl Ethers PBDEs) and PTB. While the review identified a potential link between EDC exposure and PTB, the authors argue for stronger evidence. They urge international organizations like the United Nations Environment Programme (UNEP) and the World Health Organization (WHO) to increase vigilance to curb PTB caused by EDC exposure [7].

In an animal model study, Linillos-Pradillo et al. investigated whether low-dose BPA exposure in pregnant rats would cause liver injuries in their female offspring at postnatal day 6 (PND6). Interestingly, they found that low-dose BPA exposure caused liver injury in both the lactating mothers and the female PND6 offspring. This injury appears to be mediated by increased oxidative stress, triggering an inflammatory response and apoptosis in the liver, the organ responsible for BPA detoxification. These findings suggest that BPA acts as a hepatotoxin in both pregnant rats and their female offspring [8].

Expanding the scope of this issue, Morales-Grahl et al. reviewed the adverse effects of a “new generation” of chemicals, designed to replace legacy EDCs, on brain development and behavioral disorders. They focused on early-life exposure (gestation, perinatal, and pre-puberty stages). The review highlights research on new-generation bisphenols, PFASs, and phthalates, revealing significant neurodevelopmental and behavioral alterations in zebrafish, rodents, and humans. Evidence suggests that BPA alternatives, especially BPAF, and newer PFASs, such as GenX, can significantly impact neurodevelopment and induce behavioral disorders, including IQ, autism spectrum disorders, ADHD assessments, and social behaviors in humans. The review calls for further research on phthalate replacements and bio-based alternatives, indicating these substitutes do not reduce toxicological burdens compared to legacy EDCs like BPA and certain phthalate esters [9].

Worldwide statistics indicate that the rates of over a dozen cancers are increasing among adults under 50. Models predicted a 30% increase in early-onset cancer cases between 2019 and 2030 [10]. Various factors contribute to this rise, including lifestyle factors such as EDC exposure, which may play a crucial role in early cancer development and progression. A review by Nicolella et.al. analyzed the effects of parental exposure to pesticides and other EDCs on transgenerational cancer susceptibility. The authors suggested EDC disruption of the germline epigenome as a mechanism of intergenerational inheritance [11].

Another epigenetic mechanism involved in EDC exposure and cancer development is microRNA (miRNA) dysregulation. Graziosi et al. researched the effect of low doses of atrazine, cypermethrin, and vinclozolin on miRNA dysregulation in SH-SY5Y, a human neuroblastoma cell line. They found that low-dose exposure to vinclozolin downregulated miR-29b-3p expression, leading to the increased expression of ADAM12 and CDK6 in SH-SY5Y. This promoted a pro-oncogenic response through PI3K/Akt/mTOR pathway activation, which regulates p53 activity. The discovery of miR-29b-3p as a common link for all three EDCs is of interest as this miRNA plays a significant role in neuroregeneration and may promote cancer cell survival and carcinogenesis [12].

One of the most lethal traits in cancer is the development of drug-resistant phenotypes. Some EDCs such as the per- and polyfluoroalkyl substances (PFAS), have been linked to adverse reproductive cancer outcomes in women; however, their effects on cancer progression and therapy resistance are understudied. Rickard et al. evaluated PFAS exposures in two human ovarian cancer cell lines to assess their impact on survival following carboplatin treatment. They found that PFAS co-exposure increased cell survival and affected mitochondrial function, which may explain elevated resistance to platinum drugs. The ubiquitous and persistent nature of this class of EDCs is particularly alarming among cancer patients [13].

Finally, the article by Alwadi et al. uses a risk assessment approach to show that urinary levels of environmental phenols (BPA, benzophenone-3, and triclosan) and parabens (ethyl paraben, methyl paraben, and propyl paraben) significantly correlate with self-reported prostate cancer cases among US men (The National Health and Nutrition Examination Survey, NHANES, data, 2005–2015). Using multiple gene databases and bioinformatics from many published studies on prostate cancer and toxicogenomics, the investigators identified 81 overlapping genes associated with prostate cancer and these six environmental phenols/parabens. Further analyses revealed Budding Uninhibited By Benzimidazoles 1 Homolog Beta Mitotic Checkpoint Serine/Threonine Kinase B (BUB1B), DNA Topoisomerase II Alpha (TOP2A), Ubiquitin Conjugating Enzyme E2 C (UBE2C), Ribonucleotide Reductase Regulatory Subunit M2 (RRM2), and Centromere Protein F (CENPF) to be potential markers of prostate cancer aggressiveness [14]. This study brings a new dimension to studying environmental determinants of disease using an in silico approach and big data analysis.

In aggregate, these articles have advanced our understanding of EDCs’ impact on human health and disease since our last issue [15]. By employing systematic reviews, risk assessments, adverse outcome pathways, molecular biology techniques, and bioinformatics, and focusing on low-dose effect, Developmental Origins of Health and Disease (DOHaD), and epigenetics, the evidence presented is invaluable. This research is crucial for researchers, regulatory agencies, and humanity to grasp the persistent and looming threat of EDCs to our health, both now and in future generations.
